# Enhancing Upper Limb Exoskeletons Using Sensor-Based Deep Learning Torque Prediction and PID Control

**DOI:** 10.3390/s25113528

**Published:** 2025-06-03

**Authors:** Farshad Shakeriaski, Masoud Mohammadian

**Affiliations:** Faculty of Science and Technology, University of Canberra, Canberra 2617, Australia; masoud.mohammadian@canberra.edu.au

**Keywords:** upper limb assistive exoskeleton robot elbow orthosis, torque estimation and prediction, proportional–integral–derivative control algorithm, deep learning models, stroke rehabilitation

## Abstract

Upper limb assistive exoskeletons help stroke patients by assisting arm movement in impaired individuals. However, effective control of these systems to help stroke survivors is a complex task. In this paper, a novel approach is proposed to enhance the control of upper limb assistive exoskeletons by using torque estimation and prediction in a proportional–integral–derivative (PID) controller loop to more optimally integrate the torque of the exoskeleton robot, which aims to eliminate system uncertainties. First, a model for torque estimation from Electromyography (EMG) signals and a predictive torque model for the upper limb exoskeleton robot for the elbow are trained. The trained data consisted of two-dimensional high-density surface EMG (HD-sEMG) signals to record myoelectric activity from five upper limb muscles (biceps brachii, triceps brachii, anconeus, brachioradialis, and pronator teres) during voluntary isometric contractions for twelve healthy subjects performing four different isometric tasks (supination/pronation and elbow flexion/extension) for one minute each, which were trained on long short-term memory (LSTM), bidirectional LSTM (BLSTM), and gated recurrent units (GRU) deep neural network models. These models estimate and predict torque requirements. Finally, the estimated and predicted torque from the trained network is used online as input to a PID control loop and robot dynamic, which aims to control the robot optimally. The results showed that using the proposed method creates a strong and innovative approach to greater independence and rehabilitation improvement.

## 1. Introduction

Stroke occurs when the flow of blood is obstructed or diminished in part of the brain, starving brain tissue of oxygen and nutrients needed [1]. Subsequently, neuronal injury will occur and impair brain function [2]. Depending on the severity of the stroke, individuals may suffer from different challenges, including motor function impairments, memory symptoms, attention and concentration problems, emotional fluctuations, and overall poor quality of life [3,4]. Of all these problems, motor function impairments and sequelae present the largest hurdles to completing daily tasks and becoming independent [5]. Stroke rehabilitation is therefore a crucial field of study since it helps people to recover function and improves general quality of life.

Classical rehabilitation approaches have their own advantages, however, they require more time and resources. Researchers have reduced the time and costs by improving the efficiency of rehabilitation through the development of rehabilitation robotics [6,7]. Rehabilitation robots, particularly upper limb exoskeletons, are an emerging application designed to address diverse needs of stroke survivors [8]. Exoskeletons for the upper limb are wearable robotic devices designed to assist and ease arm movement (from the shoulder to the hand). The exoskeleton allows for several movements of the arm such as reaching, grasping, and lifting, with the general intention of enhancing motor function through repetitive practice and training in functionally relevant ranges of motion. The technology offers many advantages such as range of motion improvement, coordination, reduction of pain or spasticity, and quicker recovery [9,10]. Another advantage of upper limb exoskeleton robotics is the ability to provide sensory feedback to enhance proprioception and motor control that often lacking in the conventional therapy [11]. The research on this field began over 30 years ago and achieved steady advancement in terms of design, function, and clinical features of exoskeletons [12]. Devices such as MIT-Manus, MIME, ARMGuide, and NeReBot have been able to transform rehabilitation significantly by combining mechanical assistance with therapeutic intervention [13,14,15]. Yet, despite such advancements, exoskeleton effectiveness in upper limb rehabilitation along with the ensuing research remains limited due to challenges faced by control systems [16,17,18].

One of the most important parts of an upper limb exoskeleton is the controller, which is the link between the user’s intent and the robotic movement. The design of a well-functioning controller must involve a dynamic analytical method due to the complex nature of human and robot interactions, which needs to represent user intent, accommodate user biomechanics, and operate in real-time [19]. Controllers using traditionally developed control strategies (e.g., PID and state-space control) will provide foundational elements for developing an exoskeleton, but they often fail to interpret the variability and nonlinearity in human movement and motion [20,21]. There is considerable interests in using cutting-edge techniques, particularly machine learning (ML) and artificial intelligence (AI), to enhance the flexibility, precision, and user-friendliness of these systems [22,23]. The way that exoskeletons are controlled could be transformed by incorporating ML, especially through the use of deep learning. In particular, neural networks (NNs), such as BiLSTMs and GRUs, are by nature suited for dealing with complicated and time-dependent information, making them a good match for torque and movement trajectory prediction, or intention [24]. For example, Zheng et al. proposed a Convolution-enhanced Vision Transformer model that combines convolutional and Transformer-based mechanisms for accurate lower limb exoskeleton locomotion mode recognition, achieving 98.87% accuracy for steady modes and 96.74% for transitions, outperforming traditional methods like ViT, CNN, and SVM [25]. Ren et al. developed a Transformer-based neural network that integrates convolution and variational mode decomposition to predict hip and knee joint angles from plantar force data, achieving significant improvements in gait prediction accuracy over CNN, Transformer, and hybrid CNN-Transformer models [26]. Hosseini et al. proposed a multi-modal Transformer-based model (LLMT) for real-time lower limb motion prediction in assistive robotics, achieving over 97% accuracy across various validation methods using sEMG and IMU data, and outperforming traditional machine learning and CNN models in both accuracy and prediction speed [27]. Zeng et al. proposed an sEMG-Transformer model for continuous prediction of lower limb joint angles, demonstrating superior performance over CNN, BP, and LSTM methods in enabling synchronized walking with an exoskeleton robot through accurate motion intention estimation [28]. In conjunction with model-based control strategies for robotics, such as optimal and adaptive PID control, the combination of ML and AI into the design and synthesis of hybrid controllers enables dynamic, real-time adjustments to meet user needs [29]. These hybrid systems could serve to bridge the gaps of existing control methods through the combination of mathematical model rigor and data-driven learning flexibility.

This study explores innovative control strategies for upper limb exoskeletons, emphasizing the synergy between optimal control theory and advanced deep learning techniques. We propose a creative approach to enhance the control of upper limb assistive exoskeletons by using torque estimation and prediction in a PID controller loop to more optimally integrate the torque of the exoskeleton robot, which aims to eliminate system uncertainties. Many challenges in the design of exoskeleton robot controllers, such as accurate intention recognition, real-time response, and personalization, are overcome by these methods. The aim of this paper is to improve the performance of upper limb exoskeleton robot controllers by integrating biomechanics, control theory, and artificial intelligence to create an intuitive, efficient, and effective robotic system in stroke rehabilitation.

This paper is organized as follows: Section 2 outlines the protocol for implementation with a participant, the stages of preprocessing the signals and extracting their features, the deep learning network structure involved in predicting the desired angle of the robot, the assessment of the network, the exoskeleton robot controller and its various components, which includes dynamics of the robot and evaluation of the controller. Section 3 presents the results of the experimental study on the dataset. Finally, Section 4 includes the discussion, limitations, future work, and conclusions of the research findings.

## 2. Materials and Methods

**Figure 1:**
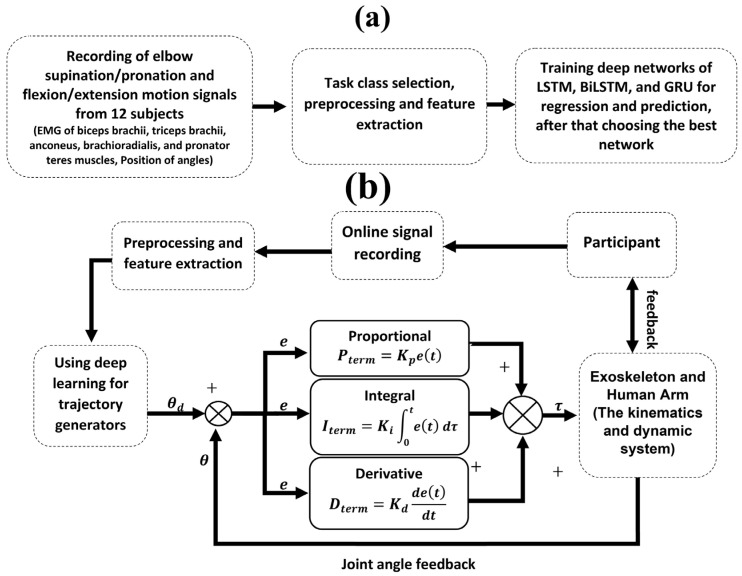
The process of performing the proposed controllers: (**a**) Training CNN, LSTM, LSTM, BiLSTM, GRU, and GRU deep neural networks for torque prediction based on sensor data; (**b**) Use best deep neural networks to predict torque-based on sensor data.

This paper presents a deep learning-based method for improving path prediction and in turn, providing assistive force estimation in an upper elbow exoskeleton robot, as knowing the future path could help with intentional coordination between the robot and the user. Deep learning models, including LSTM, BiLSTM, and GRU, are proposed and evaluated with dynamic modeling to estimate and predict the angle of the human elbow joint. These predictions serve as the basis for determining the assistive force required during rehabilitation. In the first phase of the study, publicly available data from a survey by Mónica Rojas-Martínez et al. [30] is used. The dataset includes HD-sEMG signals. Using this data, different types of neural networks are trained in two operational modes: (1) estimating the desired joint angle and (2) predicting the desired joint angle. These trained networks were then integrated into a PID control loop for an online evaluation. The aim is to evaluate the performance of each type of network in determining the trajectory of exoskeleton movement during rehabilitation tasks. Figure 1 details the research process and shows the progression from data acquisition and model training to online implementation and performance evaluation.

Classic PID controllers are usually used for linear systems, while the presented approach addresses the nonlinear aspects of the controller by using deep learning-based torque estimation and prediction. Combining PID controllers with deep learning has several significant benefits. First, Deep learning allows for compensation of nonlinearities through estimating the torque values based on the nonlinear mapping between EMG signals and joint torque. With this nonlinear mapping used within the control loop, the PID controller can provide more accurate torque inputs, meaning it can compensate uncertainty for system nonlinearities. The second benefit is that it provides a better adaptive system. Instead of simply using feedback, the PID controller now adapt based on torques predicted by the deep learning model, enabling it to be the most responsive to changes in muscle activation and movement. The final benefit of this hybrid approach is that it facilitates a path for practical application and systems stability. It is well known that PID controllers cannot necessarily work well with nonlinear systems. However, PID controllers remain the most popular option for real-time systems due to their simplicity and reliability. Deep learning builds the adaptation into a PID control while integrating all complex nonlinear aspects.

### 2.1. Dataset: Participant and Protocol Recording

**Figure 2:**
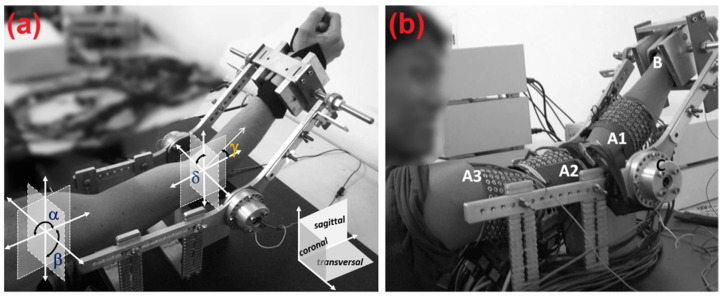
(**a**) Depiction of the upper limb’s position during the experiment, illustrating the joint angles for the shoulder and elbow; (**b**) Placement of electrode arrays 1–3 during the experiment in point of A1–A3 (Taken from the article by Mónica Rojas-Martínez et al. [30]).

The current study used publicly available data provided by Mónica Rojas-Martínez et al. [30] which involves twelve healthy participants [30]. All participants were screened to have no records of previous:(a) any neuromuscular disorders, (b) pain, or (c) regular upper limb training history while they were screened. Prior to giving their written informed consent, each participant was made fully aware of the experimental protocols and any potential risks. The study protocols adhered to ethical standards outlined in the Declaration of Helsinki and its subsequent amendments for human research. The UPC-BarcelonaTECH Ethics Committee and the local Italian Health Delivery System granted ethical approval. The dominant arm (self-reported as right for all participants) was positioned parallel to the sagittal plane with the elbow flexed at 45°, the shoulder abducted at 90°, and the forearm rotated at 90°, such that the thumb pointed upwards. Training sessions were conducted before the experiment to ensure participants could isolate forearm movements while avoiding activation of unrelated muscle groups. The wrist was stabilized using an adjustable strap and a vice at the distal end of the mechanical brace to prevent hand gripping. The experiment began with measuring Maximum Voluntary Contraction (MVC) for each task. The MVC was identified as the greatest of three successive measurements lasting roughly three seconds each. The participants were provided verbal encouragement throughout the MVC measurements to inspire the athlete to give their maximum effort. There was a two-minute recovery time between trials to minimize fatigue. These tasks included supination, pronation, elbow flexion, and elbow extension. After determining their MVC, the participants completed isometric contractions at 10%, 30%, and 50% of their MVC for each item. Each task was to be completed for 10 s each, with two minutes of rest between each contraction to avoid cumulative fatigue. The order of each task was randomized to eliminate any potential order effects. An instructor was present during each trial to specifically monitor technical performance of the functions. Multi-electrode grids were used to capture high-density surface EMG signals at a sampling rate of 2048 Hz over five different upper limb muscles: the biceps brachii (BIC), triceps brachii (TRI), anconeus (ANC), brachioradialis (BRD), and pronator teres (PRT). Signal calibration and preprocessing were completed rigorously to minimize collected noise and artifacts, and ensure signal quality. The controlled environment and standardized protocols ensured reproducible data for neuromuscular analysis and computational modeling. Demographic information of the participants is presented in Table 1, and details of the experimental setup and procedure are illustrated in Figure 2.

### 2.2. Preprocessing and Feature Extraction

The dataset consists of electromyography (EMG) sensor readings and elbow joint torque measurements, which were initially converted from bin to mat format for analysis. EMG signals went through a preprocessing stage to confirm the quality of the signal. This preprocessing includs a notch filter at 50 Hz to eliminate powerline interference, high-pass filtering at 5 Hz to reduce motion artifacts and DC offsets, and low-pass filtering at 500 Hz to attenuate high frequency noise. All the filters used the minimum filter order and damping to not alter the signal’s integrity. EMG signals from each two-dimensional plane were averaged to generate representative signals for analysis, resulting in three final EMG signals. Fourteen time-domain pattern recognition algorithms were utilized to extract relevant features from the EMG signals. These features, detailed in the Supplementary Materials, include Root Mean Square, Waveform Length, Mean Absolute Value, and others. The selection of these features is based on their ability to capture different aspects of muscle activation, amplitude-based features (e.g., Root Mean Square, Mean Absolute Value, Simple Square Integral) quantify the overall muscle activation level, providing a direct correlation with torque generation, frequency and signal complexity features (e.g., Zero Crossing, Slope Sign Change, Willison Amplitude) reflect muscle contraction dynamics and motor unit recruitment patterns, and variability and statistical features (e.g., Standard Deviation, Difference in Absolute Standard Deviation Value) indicate changes in muscle activation over time. A sliding window approach with a length of 100 samples and a 99-sample overlap is applied to extract these features continuously over time, allowing detailed pattern recognition. A buffer window with 200 samples is applied to the torque signals to ensure smooth data representation. This step mitigates noise fluctuations and aligns the torque measurements with EMG-derived features for improved predictive modeling.

### 2.3. Deep Model for Regression and Prediction

Three deep learning architectures LSTM, BiLSTM, and GRU are implemented to perform regression and prediction tasks using HD-sEMG signals as inputs. The models are designed to estimate and predict the angle of the human elbow joint to determine assistive forces in a one degree of freedom (DOF) upper-limb exoskeleton robot. The evaluation consisted of a regression model, and an angle estimation model which used similar architectures. The individual architecture for the three forms of models is as follow:

- Input Layer: The input is the features of the preprocessed HD-sEMG signals based on time domain analysis.
- Recurrent Layers: Consist of LSTM, BiLSTM, or GRU depending on the architecture. These layers capture temporal dependencies in the data and encode sequential information.
- Fully Connected Layers: A dense layer maps the output of the recurrent layers to the regression or prediction output.
- Output Layer: Produces the estimated or predicted joint angle as a single continuous value.

The input sequence $X\in R^{T\times N}$ consists of T timesteps and N features derive from the HD-sEMG signals, where T is the length of the input window, and N is the number of features.

- LSTM Model: The LSTM model utilizes memory cells to retain long-term dependencies [31,32,33]. The cell state $C_{t}$ and hidden state $h_{t}$ are updated at each timestep as follows:

(1) $$f_{t}=\sigma \left(W_{f}\cdot \left[h_{t-1},x_{t}\right]+b_{f}\right)$$

(2) $$i_{t}=\sigma (W_{i}\cdot [h_{t-1},x_{t}]+b_{i})$$

(3) $${\tilde{C}}_{t}=\tanh\left(W_{C}\cdot \left[h_{t-1},x_{t}\right]+b_{C}\right)$$

(4) $$C_{t}=f_{t}⊙C_{t-1}+i_{t}⊙{\tilde{C}}_{t}$$

(5) $$o_{t}=\sigma (W_{o}\cdot [h_{t-1},x_{t}]+b_{o})$$

(6) $$h_{t}=o_{t}⊙\tanh\left(C_{t}\right)$$

Here, $f_{t}$, $i_{t}$, $o_{t}$ represent the forget, input, and output gates, respectively. σ is the sigmoid activation function, and ⊙ denotes element-wise multiplication.

- BLSTM Model: The BiLSTM model extends LSTM by processing the input sequence in both forward and backward directions, capturing bidirectional dependencies [34,35]. The hidden states from both directions are concatenated as follows:

(7) $${\vec{h}}_{t}=LSTM\left(x_{t},{\vec{h}}_{t-1}\right)$$

(8) $${\overset{\leftarrow }{h}}_{t}=LSTM\left(x_{t},{\overset{\leftarrow }{h}}_{t-1}\right)$$

(9) $$h_{t}=\left[{\vec{h}}_{t};{\overset{\leftarrow }{h}}_{t}\right]$$

- GRU Model: The GRU model simplifies the LSTM by combining the forget and input gates into an update gate $z_{t}$ and using a reset gate $r_{t}$ to modulate the input [36,37]. The updates are given by:

(10) $$z_{t}=\sigma \left(W_{z}\cdot \left[h_{t-1},x_{t}\right]+b_{z}\right)$$

(11) $$r_{t}=\sigma \left(W_{r}\cdot \left[h_{t-1},x_{t}\right]+b_{r}\right)$$

(12) $${\tilde{h}}_{t}=\tanh\left(W_{h}\cdot \left[r_{t}⊙h_{t-1},x_{t}\right]+b_{h}\right)$$

(13) $$h_{t}=\left(1-z_{t}\right)⊙h_{t-1}+z_{t}⊙{\tilde{h}}_{t}$$

- Loss Function: For both regression and prediction models, the loss function used is the Mean Squared Error (MSE) [32,38], defined as:

(14) $$L=\frac{1}{n}\sum_{i=1}^{n}{\left(y_{i}-{\hat{y}}_{i}\right)}^{2}$$

where $y_{i}$ is the true angle, ${\hat{y}}_{i}$ is the predicted angle, and n is the number of samples.

**Figure 3:**
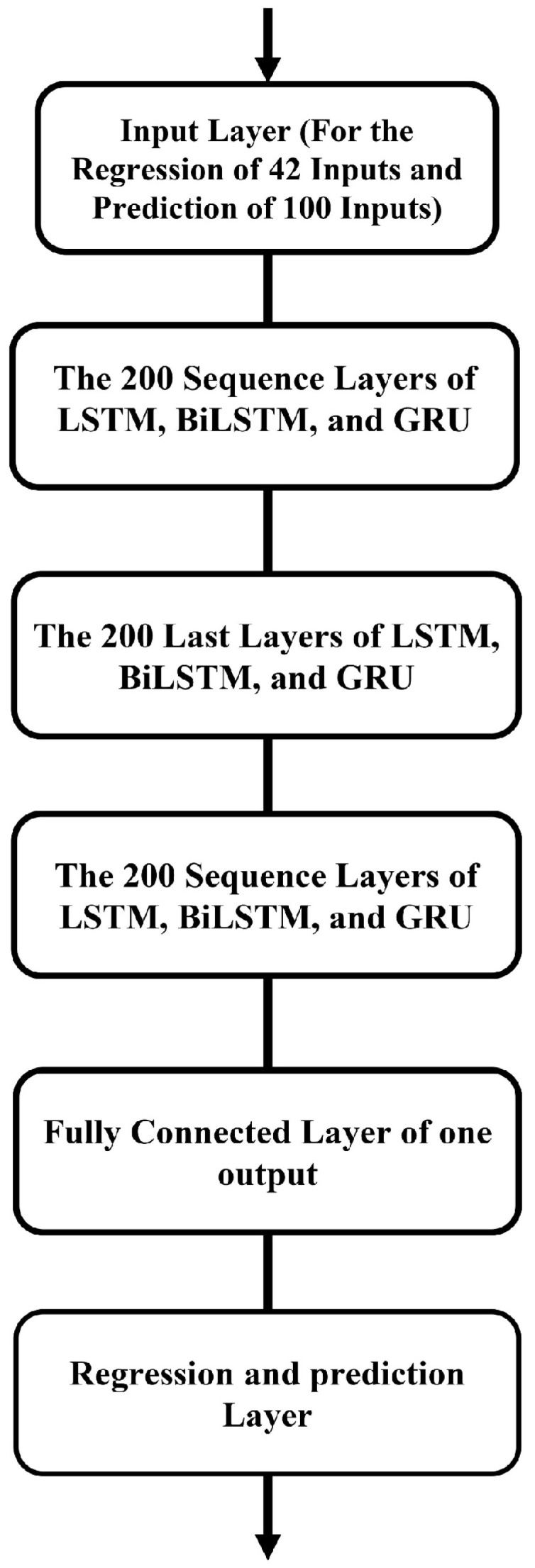
Proposed architecture of neural networks for robot position prediction and reaction.

Since three EMG signals are extracted at each instant and 14 patterns are identified from each, the regression architecture processes 42 inputs to produce the robot’s desired angle as the output. For the predictive architecture, the input consists of data from the 100 preceding frames of angle, collected 200 samples earlier, corresponding to an estimation time of 410/2048 = 0.2 s earlier. An initial architectural search was performed by varying the number of hidden layers (1–3) and the number of units per layer (32, 64, 128), and we arrived at the final network architecture model in Figure 3. The network training employed the Adam optimizer with a comprehensive set of hyperparameters to achieve efficient and effective learning. The training procedure is set for 1000 epochs, in order to provide an ample amount of iterations for convergence. A gradient threshold of 1 is utilized to manage gradient issues and avoid occurrences of gradient explosion. An initial learning rate of 0.005 and a piecewise learning rate schedule promotes a gradual optimization process for the learning rate. This implies that after 125 epochs, the learning rate would de-crease by a factor of 0.2, facilitating smaller weight updates during the training process. A mini-batch size of 120 is selected as the target mini-batch size in order to make the trade-off between computation and performance, and computation on a GPU is specifically chosen to take advantage of accelerated computing. Additionally, the training progress is visually monitored by plots, for the sake of transparency through-out the task. Hyperparameters are adequately chosen so that the performance of the network architecture is maximized while allowing for the successful creation of the model.

### 2.4. Performance of Evaluation Deep Learning Model

To assess the performance of the deep learning models, three metrics are used: MSE, Root Mean Squared Error (RMSE), and Pearson Correlation Coefficient (PCC). These metrics are selected to evaluate the accuracy, consistency, and correlation between the predicted and actual joint angles.

- Mean Squared Error: MSE measures the average squared difference between the predicted (${\hat{y}}_{i}$) and actual ($y_{i}$) values. It is defined as:

(15) $$MSE=\frac{1}{n}\sum_{i=1}^{n}{\left(y_{i}-{\hat{y}}_{i}\right)}^{2}$$

where n is the number of samples. A lower MSE value indicates a better fit of the model to the data.

- Root Mean Squared Error: RMSE is the square root of the MSE, providing a measure of the error in the same units as the target variable [39]. It is given by:

(16) $$MSE=\sqrt{\frac{1}{n}\sum_{i=1}^{n}{\left(y_{i}-{\hat{y}}_{i}\right)}^{2}}$$

RMSE is more interpretable than MSE and is sensitive to large errors, making it suitable for evaluating the accuracy of predictions.

- Pearson Correlation Coefficient: PCC quantifies the linear correlation between the predicted and actual values [40]. It is defined as:

(17) $$PCC=\frac{\sum_{i=1}^{n}\left(y_{i}-\bar{y}\right)\left({\hat{y}}_{i}-\bar{\hat{y}}\right)}{\sqrt{\sum_{i=1}^{n}{\left(y_{i}-\bar{y}\right)}^{2}\sum_{i=1}^{n}{\left({\hat{y}}_{i}-\bar{\hat{y}}\right)}^{2}}}$$

where $\bar{y}$ and $\bar{\hat{y}}$ are the means of the actual and predicted values, respectively. PCC ranges from −1 to 1, where 1 indicates a perfect positive linear correlation, 0 indicates no correlation, and −1 indicates a perfect negative linear correlation.

MSE and RMSE give a sense of the size of prediction errors. PCC quantifies the strength and the direction of association between predicted and actual values. These complementary metrics provide a holistic evaluation of the models with respect to accuracy and consistency in predicting joint angles. The findings from these metrics of model performance are presented in the Results section, with details of the performance of LSTM, BiLSTM, and GRU models in each of the regression and forecasting tasks.

### 2.5. Dynamics of the Human-Robot System

**Figure 4:**
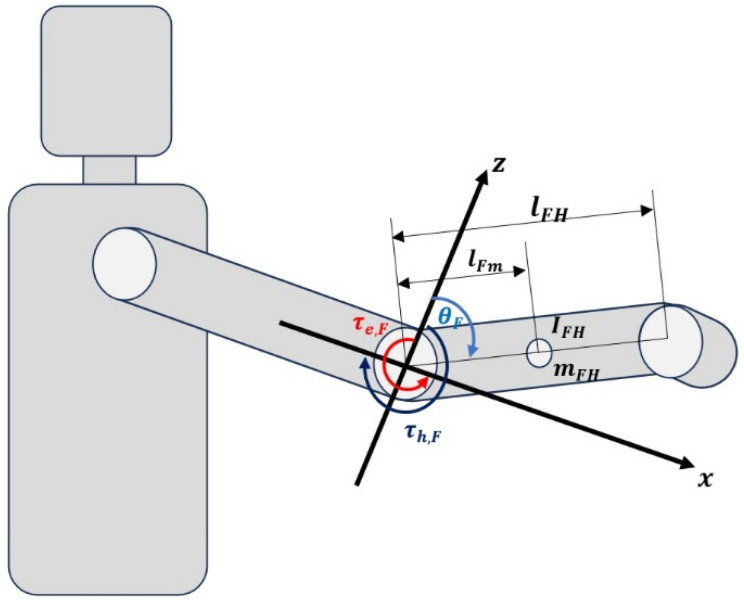
Dynamic model of the elbow orthosis robot of the auxiliary exoskeleton of the upper limb, one degree of freedom.

Figure 4 depicts the dynamic model of a single-degree-of-freedom upper limb assistive exoskeleton robot elbow orthosis. The limb’s movement is confined to the sagittal plane (x−z). The central axis (0,0) represents the elbow joint, with its physical parameters illustrated in the figure. Key parameters include inertia $I_{FK}$, mass $m_{FK}$, link length $l_{FK}$, link length from joint to center of mass $l_{Fm}$, and the angle $\theta_{F}$ of the forearm joint relative to the (x−z) coordinates, where $\theta_{F}\epsilon R^{n}$. The torque applied by the user to the system is denoted as $\tau_{h,F}$, and $\tau_{l,F}$ represents the joint torque from motors. This study uses the Euler-Lagrange method to describe the dynamic model of the exoskeleton robot’s one-degree-of-freedom forearm joint [41]. The coordinates $O_{Forearm}\left(x_{Forearm},y_{Forearm}\right)$ and $O_{Hand}\left(x_{Hand},y_{Hand}\right)$ denote the centers of the forearm and hand parts, respectively. The final position of the robot is given by:

(18) $$\left\{\begin{array}{c} x_{Hand}=l_{Fm}\sin\left(\theta_{F}\right)\\ y_{Hand}=l_{Fm}\cos\left(\theta_{F}\right) \end{array}\right.$$

In the Lagrangian formulation, the Lagrangian of the total system is obtained by the total kinetic energy minus the total potential energy of the discharge, the total kinetic energy (T) of the system is defined as follows:

(19) $$T=\frac{1}{2}\left(m_{FH}l_{Fm}^{2}{\dot{\theta }}_{H}^{2}+I_{FH}{\dot{\theta }}_{F}^{2}\right)$$

The potential energy V of the system due to gravity is:

(20) $$V=m_{FH}gl_{Fm}\sin\left(\theta_{F}\right)$$

The Lagrangian L of the entire system is:

(21) $$L=T-V=\frac{1}{2}\left(m_{FH}l_{Fm}^{2}{\dot{\theta }}_{F}^{2}+I_{FH}{\dot{\theta }}_{F}^{2}\right)-m_{FH}gl_{Fm}\sin\left(\theta_{F}\right)$$

From the Lagrangian expression, the equation of motion for the entire system is derived as:

(22) $$\frac{d}{dt}\left(\frac{\partial L}{\partial \dot{\theta }}\right)-\frac{\partial L}{\partial \theta }=\tau_{l}+\tau_{h}$$

where the partial derivatives are:

(23) $$\frac{\partial L}{\partial \dot{\theta }}=m_{FH}l_{Fm}^{2}{\dot{\theta }}_{F}+I_{FH}{\dot{\theta }}_{F}$$

(24) $$\frac{d}{dt}\left(\frac{\partial L}{\partial \dot{\theta }}\right)=m_{FH}l_{Fm}^{2}{\ddot{\theta }}_{F}+I_{FH}{\ddot{\theta }}_{F}$$

(25) $$\frac{\partial L}{\partial \theta }=-m_{FH}gl_{Fm}\cos\left(\theta_{F}\right)$$

Here, ${\dot{\theta }}_{F}$ denotes angular velocity and ${\ddot{\theta }}_{F}$ denotes angular acceleration of the forearm joint.

The dynamic equation of the robot is thus formulated as:

(26) $$\left(m_{FH}l_{Fm}^{2}+I_{FH}\right){\ddot{\theta }}_{F}+m_{FH}gl_{Fm}\cos\left(\theta_{F}\right)=\tau_{l}+\tau_{h}$$

### 2.6. Proportional Integral Derivative Controller

In this study a PID control is used to provide upper limb rehabilitation treatment. PID controller is the most widely used control technique in industrial applications. The PID controller is simple in design and efficient in calculations. In addition, a robust control technique is considered. The following equation can express the outline of the PID control approach:

(27) $$\tau_{h}=K_{P}\left(\theta_{d}-\theta \right)+K_{I}\int \left(\theta_{d}-\theta \right)dt+K_{D}\left({\dot{\theta }}_{d}-\dot{\theta }\right)$$

(28) $$\tau_{h}=K_{P}\left(\theta_{d}-\theta \right)+K_{I}\int \left(\theta_{d}-\theta \right)dt+K_{D}\left({\dot{\theta }}_{d}-\dot{\theta }\right)$$

where $\theta_{d}$, θ are the desired and measured joint angle vectors, respectively [42].

### 2.7. Performance of Controller

To evaluate the performance of the controller when neural network models are online, two metrics were employed: Normalized Root Mean Square Error (NRMSE) and Mean Error (ME). These metrics are selected to measure the precision and overall accuracy of the controller’s output in real-time tasks.

- Normalized Root Mean Square Error (NRMSE): NRMSE is a scaled version of RMSE, normalized by the range or mean of the actual values, providing a dimensionless measure of error [43]. It is defined as:

(29) $$NRMSE=\frac{\sqrt{\sum_{i=1}^{n}\frac{{\left(y_{i}-{\hat{y}}_{i}\right)}^{2}}{n}}}{y_{max}-y_{min}}$$

where, $y_{i}$ and ${\hat{y}}_{i}$ are the actual and predicted values, respectively. And $y_{max}$ and $y_{min}$ are the maximum and minimum values of the actual data. NRMSE provides a percentage-like representation of error, making it easier to interpret across different scales. Lower values of NRMSE indicate better controller performance.

- Mean Error (ME): ME measures the average deviation of the predicted values from the actual values. It is calculated as:

(30) $$ME=\frac{1}{n}\sum_{i=1}^{n}\left(y_{i}-{\hat{y}}_{i}\right)$$

ME can indicate any systematic bias in the controller’s performance, with positive values showing an overestimation and negative values an underestimation of the target.

NRMSE assesses the relative accuracy of the controller output taking into account the range of actual values. ME provides information on the average deviation and shows any systematic offsets in the control performance. These metrics are used to evaluate how the controller performed in tracking the desired path and maintaining the correct joint angles in real-time applications. The evaluation results demonstrate that the proposed models are suitable for the control tasks in addition to supporting performance in different conditions.

### 2.8. Statistical Analysis

We conducted a comprehensive statistical analysis to assess the significance of performance differences among models and under varying SNR conditions. Prior to applying inferential tests, the data distributions for each performance metric—namely RMSE, correlation coefficient, and trajectory tracking error—were evaluated for normality using the Shapiro–Wilk test. Homogeneity of variances across groups was assessed using Levene’s test. Upon satisfying the assumptions of normality and equal variances, a one-way analysis of variance (ANOVA) was performed to determine whether the observed differences in performance metrics across experimental conditions were statistically significant, where ANOVA indicated significant differences (p<0.05). A threshold of p<0.05 was considered statistically significant throughout the analysis. All significant comparisons are explicitly marked in the revised result tables and figures using asterisks and appropriate annotations.

## 3. Results

The performance of three deep learning algorithms—LSTM, BiLSTM, and GRU—was evaluated on two network architectures designed for regression and prediction tasks. For training and validation, 70% of the total dataset was randomly selected, while 30% was reserved for testing. The robustness of the models is tested by introducing white Gaussian noise at specific SNR levels (0, 2, 5, 10, and 20 dB) into the training signals. The SNR levels are explicitly measured in decibels (dB), as now indicated in Table 2 and Table 3. These values are selected based on typical signal quality observed in EMG recordings, where noise contamination stems from motion artifacts, electrode-skin impedance variations, and environmental interference. Performance is measured using the: MSE, RMSE, and PCC, summarized in the tables below for the regression network (Table 2) and the predictive network (Table 3).

Table 2 compares how three recurrent networks—LSTM, bidirectional LSTM (BLSTM), and GRU—cope with progressively cleaner HD-sEMG input when estimating elbow-joint torque. White-Gaussian noise is injected at six signal-to-noise ratios (SNR = 0, 1, 2, 5, 10, 20 dB); mean-squared error (MSE) and its root (RMSE) are reported on both the training set and an unseen test set. Under the heaviest noise (0 dB), the GRU achieves the lowest training RMSE (1.28 N·m), followed by the LSTM (1.40 N·m) and the BLSTM (1.50 N·m). This ranking reflects architectural capacity: the GRU’s simpler gate structure can over-adapt to random fluctuations and minimizes in-sample error. Yet the picture changes on unseen data: at 0 dB, the LSTM and GRU tie for best test RMSE (3.49 N·m), while the BLSTM edges slightly ahead (3.37 N·m) at 1 dB. These small shifts suggest that bidirectional context helps when forward information is almost obliterated, but becomes less valuable as soon as a modest signal emerges. At moderate noise levels (2 dB and 5 dB), the LSTM–BLSTM pair trades the lead. The BLSTM posts the lowest test RMSE at 2 dB (3.44 N·m) thanks to its look-ahead advantage. Still, at 5 dB the GRU unexpectedly resurfaces with the best score (3.46 N·m), hinting that a lighter model can generalize well once the signal-to-noise ratio crosses a threshold. Nevertheless, the LSTM remains within 0.13 N·m of the leader at every SNR, underscoring its stability. As noise continues to recede (10 dB and 20 dB) all three networks converge: test RMSEs lie between 3.55 N·m and 3.65 N·m, and the LSTM is marginally superior. Flattening all test-error curves implies that model choice becomes less critical when the sEMG signal is already clean; what distinguishes the architectures is their behavior in the more challenging, low-SNR regime. Two general conclusions emerge. First, the LSTM offers the most consistent generalization: it is never the worst performer and often the best, with a remarkably flat error profile (±0.13 N·m across the entire SNR sweep). Second, the GRU’s train–test gap is the largest at 1 dB and 2 dB, confirming that its excellent training scores stem partly from noise memorization. The BLSTM’s bidirectionality yields sporadic gains but no sustained advantage, and in real-time control, a bidirectional network introduces latency or must be truncated, reducing its practical utility. For a hybrid PID exoskeleton controller that may encounter fluctuating SNR daily use, robustness matters more than peak accuracy at one specific noise level. Table 2, justifies adopting the LSTM-based torque estimator: it balances resilience to extreme noise, resistance to over-fitting, and consistent performance when the signal cleans up—qualities that translate into smoother, more reliable joint-angle tracking in the closed-loop system. The performance of Table 3 was similar to that of Table 2.

**Figure 5:**
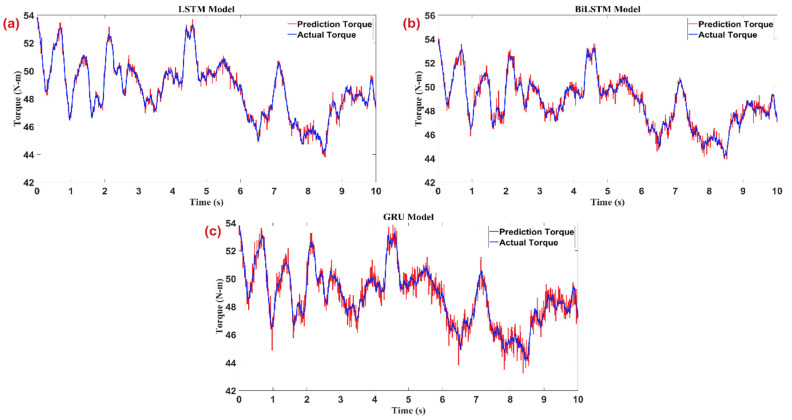
Comparison of predicted and actual torque signals over time for (**a**) LSTM, (**b**) BLSTM, and (**c**) GRU deep learning models. The results demonstrate the effectiveness of the trained networks in accurately capturing the temporal dynamics of the exoskeleton joint torque signals.

Figure 5 portrays how three recurrent neural-network architectures—LSTM, Bi-LSTM, and GRU—track elbow-joint torque over a ten-second interval when the input HD-sEMG is noise-free. Although the three panels share identical axes (≈42–56 N·m vertically, 0–10 s horizontally), each model leaves a distinct visual signature that reveals its strengths and weaknesses. The LSTM prediction (red) almost merges with the upper-left panel’s ground-truth torque (blue). Phase alignment is exact: every peak, trough, and inflection co-occurs, and the amplitude error seldom exceeds half a Newton-meter. The smooth trace indicates that the LSTM’s gating mechanism suppresses the inevitable acquisition noise without blunting fast transients. Consequently, the LSTM can be described as both precise and well-damped. The Bi-LSTM in the upper-right panel tells a slightly different story. Because the network consumes information in both temporal directions, it “anticipates” sharp reversals and occasionally overshoots them. This manifests as brief red spikes above blue peaks around 4.8 s and again near 7.4 s. Otherwise, the fit is tight; the bidirectional context improves continuity in regions where the torque signal oscillates rapidly. This anticipation would be advantageous for an off-line analysis task, but in a real-time controller, it introduces minor amplitude errors that the PID loop must dissipate. The lower panel, showing the GRU, reveals the trade-off that comes with architectural parsimony. The red curve follows the overall envelope of the true torque, but a slight lag is noticeable during the steepest slopes, and the high-frequency jitter is visibly larger. Peaks are rounded off and valleys are underestimated, suggesting that the GRU’s simpler gating cannot capture the full dynamic range without introducing bias. While this model would consume less memory and inference time on an embedded processor, its higher instantaneous error would force the PID controller to work harder, potentially leading to larger corrective torques and less stable actuation. The three panels substantiate the manuscript’s quantitative rankings: LSTM yields the smallest root-mean-square error, Bi-LSTM is a close second with occasional overshoot, and GRU, though computationally efficient, delivers the least accurate reconstruction. Visually confirming these differences is important because it demonstrates that the proposed hybrid scheme is statistically superior and qualitatively better in reproducing the fine temporal structure of human-generated torque.

**Figure 6:**
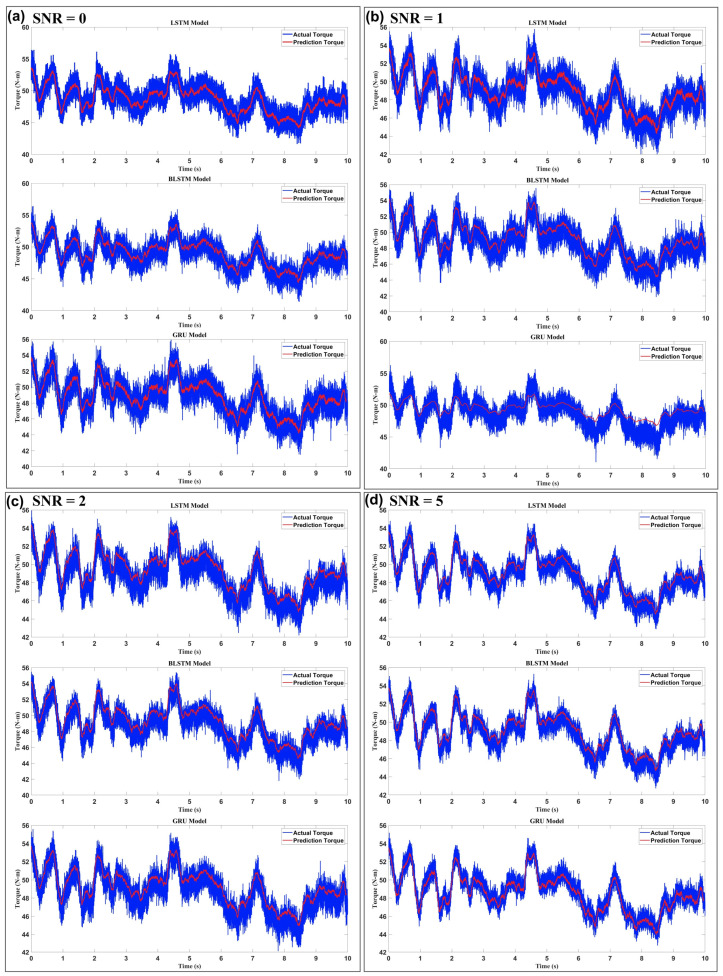

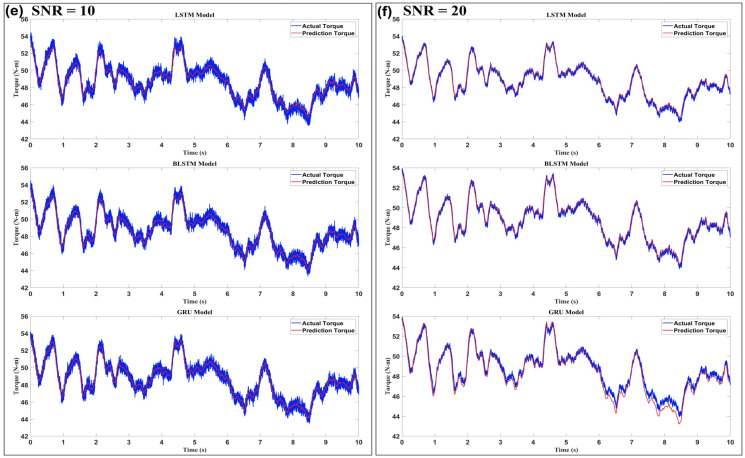
Evaluation of model robustness under varying levels of white Gaussian noise (SNR) ((**a**) 0, (**b**) 1, (**c**) 2, (**d**) 5, (**e**) 10, and (**f**) 20). The plots illustrate the impact of noise on the prediction accuracy of the LSTM, BLSTM, and GRU models, highlighting their generalization capabilities in noisy environments.

Figure 6 illustrates how three recurrent neural-network architectures—LSTM, bidirectional LSTM (Bi-LSTM), and GRU—cope with progressively cleaner high-density sEMG input when estimating elbow-joint torque. Each SNR level occupies its column, ranging from 0 dB (the harshest noise) to 20 dB (virtually noise-free). The models are stacked vertically within every column, and, crucially, all panels share identical axes (42 –56 N·m on the ordinate and a ten-second time window on the abscissa). This consistent scaling allows a direct visual comparison of absolute error and dynamic-range coverage. At the lowest SNR of 0 dB, the blue ground-truth torque trace is completely enveloped in wide-band noise, yet the red prediction curves still shadow its overall shape. Among the three networks, the LSTM delivers the narrowest prediction envelope; its red trajectory rarely strays more than ~2 N·m from the blue reference, whereas the GRU exhibits noticeably larger jitter and occasional under- or overshoot on sharp peaks. The Bi-LSTM falls in between, displaying mild overshoot that can be attributed to its bidirectional context anticipating forthcoming signal changes without entirely suppressing the noise. The gap between networks shrinks as the SNR climbs to 1 dB and 2 dB. Still, the rank ordering remains: the LSTM produces the cleanest overlay, Bi-LSTM is only marginally noisier, and the GRU continues to lag, especially in valleys were torque transitions rapidly. A qualitative threshold becomes visible at 5 dB. Beyond this point, the LSTM and Bi-LSTM prediction traces hug the reference so tightly that only minor ripples betray their estimation error. At the same time, the GRU still shows a faintly wider band—evidence that its simpler gating mechanism is less effective at filtering residual noise. By 10 dB, the distinction is subtler still. All three networks reproduce amplitude and phase with high fidelity; their instantaneous absolute errors rarely exceed half a Newton-meter. Nevertheless, a close inspection reveals that the GRU slightly compresses extreme peaks, whereas the LSTM and Bi-LSTM follow them almost point-for-point.

Finally, at 20 dB, the three red traces become practically indistinguishable from the blue baseline; the remaining discrepancies lie well inside the motor-control tolerance of a typical elbow orthosis. Taken together, Figure 6 illustrates key phenomena. First, prediction accuracy improves monotonically with SNR. Still, the improvement is not linear: an enormous performance leap occurs between 2 dB and 5 dB, implying that sEMG features critical for torque decoding emerge from the noise floor within this range. Second, architectural resilience differs. The LSTM consistently exhibits the best noise immunity, the Bi-LSTM offers nearly equivalent performance except for a tendency toward slight overshoot at very low SNRs, and the GRU, while computationally lighter, pays a penalty in both high-frequency jitter and slight amplitude bias. These visual findings align with quantitative metrics reported elsewhere in the manuscript (e.g., RMSE and correlation coefficients) and justify the choice of the LSTM as the torque estimator feeding the hybrid PID loop in the proposed exoskeleton control framework.

## 4. Discussion and Conclusions

The proposed work utilized advanced deep learning techniques to introduce a novel approach for estimating and predicting human joint angles and associated assistive forces for a 1 DOF upper exoskeleton robot elbow. The system utilized a powerful combination of LSTM, BiLSTM, and GRU with dynamic modeling to leverage HD-sEMG signals for real-time torque value prediction and future movement anticipation. The signal extraction process was reliable and accurate using preprocessing methods such as multi-stage filtering and dimensionality reduction that provided inputs with high confidence to the learning models. Furthermore, fourteen time series pattern recognition algorithms developed a greater depth of understanding myoelectric for mapping EMG signals with a close estimation of joint torque. The developed system outperformed classical methods in terms of generalizability, adaptability, implementation in real-time, and robustness, ultimately improving the system’s application in human-robot interaction in rehabilitation and assistive technology.

Although BiLSTM can capture bidirectional temporal dependencies and GRU has computational advantages, LSTM was superior in the case study. This can be due to the reasons expressed. First, EMG-to-torque estimation in real-time scenarios is primarily dependent on identifying and estimating torque based on past observations. Future context will be irrelevant in the prediction of torque, as there is no future data for EMG signals to be included. Since BiLSTM processes data from two directions, its bidirectional nature affords it no explicit advantage in this context for EMG-to-torque prediction. Second, model complexity matters. BiLSTM has approximately double the number of parameters that LSTM has, which could increase the potential of overfitting, particularly when considering the dataset size. Although BiLSTM would have the same capacity as LSTM for capturing long-term dependencies, it does so at the cost of increased parameterization. By comparison, LSTM captures meaningful long-term dependencies in muscle activation while managing the parameters of complexity and learning capabilities. Finally, the results shows that LSTM produced more stable training, with improved generalization for a torque estimation task. While GRU is faster to train and less complex than LSTM, it may have been less able to capture long-term patterns of muscle activation and, therefore, resulting in lower predictive performance. Overall, it is found that although BiLSTM and GRU have their strength in differing contexts, LSTM was an overall more suitable model for real-time EMG-to-torque predictions because it was able to capture significant dependencies while balancing the complexity of the model [44,45,46,47].

In order to evaluate the improvements in performance achieved through the deep learning method for torque estimation, it was compared with a baseline condition in which a standard PID controller had been implemented. The PID baseline used fixed motion inputs, without any estimation properties derived from learning. An important distinction between the deep learning strategy employed and a standard PID lay with respect to propulsion or prediction capabilities of each type of controller. The baseline PID controller only has feedback in real-time and, therefore, is more subject to delays in system and to disturbances. The integrated learning-based model analyzes patterns in previous sensor data to gain an acceptable understanding of anticipated joint motion prior to the command, which improved responsiveness and stability. Moreover, the absence of learning proposed by the conventional system limits the degree of adaptation, leading to a somewhat rigid closed-loop control. On the other hand, the presented model adapts torque estimates according to EMG engagement rate and as previous EMG changed in real time—making the model adaptable. In conclusion, the testing demonstrated smooth trajectory response, reduced torque variation, and user comfort for a deep learning-adjusted PID controller—all important attributes in rehabilitation when user-friendly and adaptable personalized motion-control is essential [48,49].

The baseline comparison highlights the noted performance benefits from utilizing deep learning, thus emphasizing the importance of the presented hybrid control strategy. While the deep learning model achieved high accuracy for torque estimation, it poses challenges in deployment for real-time applications. One of the challenges is computational latency, and thus an evaluation of the inference times for each model-LSTM, BiLSTM and GRU were provided and it was identified that LSTM generates a better balance between accuracy and speed. Strategies that considered to reduce computational latency included using smaller batch sizes for training and possibly model pruning [50]. The second challenge is due to hardware constraints; typically, real-time controllers need to deploy deep leaning models on embedded systems which usually have a limit in computational power, memory and energy use. Also, the results indicated the possibility of running LSTM based torque estimation on edge devices, and some options were considered for hardware acceleration as well. Finally, there is a trade-off between accuracy and latency/speed which were addressed through options like quantization, knowledge distillation, and FPGA based acceleration to allow for real-time deployments with acceptable predictive performance yet lower latency.

The proposed work has limitations despite its advantages. The research used public data, which, while adequately researched, still may not include some of real-world variability (e.g., type of subjects, muscle conditions, and activities beyond isometric contractions). Furthermore, using a fixed window length and overlapping in the preprocessing stage, while effective, could introduce constraints in tasks requiring more dynamic adaptability. The computational complexity of the deep learning models and the need for precise calibration may pose challenges in deploying this system on low-power or embedded hardware in real-world applications. Additionally, the study focused on a single DOF, which limits its generalizability to more complex, multi-DOF movements commonly encountered in daily activities or advanced rehabilitation scenarios.

The proposed deep learning-based torque estimation and control framework was developed and validated using data collected from healthy subjects. Nonetheless, the natural differences were recognized in aspects of neuromuscular control and movement patterns when comparing healthy individuals to patients with stroke. Patients with stroke tend to have muscle weakness, spasticity, and diminished motor coordination; thus, these factors may directly result in differences in the characteristics of the electromyography (EMG) signal and torque patterns for patients with stroke compared to healthy individuals. These factors introduce additional challenges when directly applying the current model to stroke rehabilitation scenarios. To address this limitation, leveraging transfer learning was proposed to adapt the deep learning model to patient-specific characteristics. Transfer learning enables pre-trained models to be fine-tuned with a smaller dataset collected from stroke patients, allowing the system to account for variations in muscle activation and movement control. This method decreases the demand for extensive retraining while increasing the model’s generalized properties to the clinical population. In addition, adaptive filtering of the EMG inputs and learned patient calibration approaches could enhance the robustness of the torque estimation and allow the model to account for patient-dependent variation in the neuromuscular response.

This work highlights the potential of advanced deep learning techniques and HD-sEMG signal analysis in achieving accurate estimation and prediction for assistive robotics. The work offers a strong basis for future developments in human-robot cooperation and rehabilitation technology by showing the superiority of LSTM in real-time torque estimation and combining deep learning with PID control, and the goal of future work is to implement this model on hardware. In future work, we plan to investigate the use of transformer-based architectures for torque prediction in upper-limb exoskeletons, given their proven ability to capture long-range temporal dependencies and contextual information more effectively than traditional RNNs. Additionally, we aim to explore lightweight edge-AI techniques such as model pruning, quantization, and TinyML to enable real-time implementation on low-power embedded platforms. These directions hold promise for improving both the performance and practical deployment of wearable robotic systems, and can contribute to more responsive and portable solutions for assistive and rehabilitation applications.

## Figures and Tables

**Table 1:** Demographic information of the individuals whose data is recorded.

Subject	S1	S2	S3	S4	S5	S6	S7	S8	S9	S10	S11	S12	*p*-Value
Age (years)	28	29	21	31	28	22	22	27	28	40	29	37	≈0
Height (cm)	176	183	183	175	176	187	175	171	168	178	173	179	≈0
Weight (kg)	76	90	83	68	70	82	72	68	75	83	64	65	≈0
Biceps circumference (cm)	31	36	37	30.5	29	32	33	34	32.5	33	31	29	≈0
Biceps length (cm)	33	33	33.5	30	30	30	31	34	30	32.5	31.5	32	≈0
Triceps circumference (cm)	31	34	34	29	28	30	30	30	34	34	29	29.5	≈0
Triceps length (cm)	40	39	41	34	34	40	38	37	37	37.5	36.5	37	≈0
Forearm circumference (cm)	26	32	32	27	20	28	27	29	27	28	26.5	27	≈0
Forearm length (cm)	30	30	30	29	28	31	29	30	28	26.5	25	28.5	≈0

**Table 2:** Robustness evaluation of regression models (LSTM, BLSTM, GRU) under varying levels of white Gaussian noise (SNR). Unlike time series prediction, which focuses on forecasting future values based on temporal dependencies, this regression task aims to estimate continuous-valued outputs from input features at each time step. Performance is assessed using MSE, RMSE, and PCC across different SNR conditions to examine each model’s resilience to noisy input data.

		Evaluation Metrics	SNR 0 dB	SNR 1 dB	SNR 2 dB	SNR 5 dB	SNR 10 dB	SNR 20 dB
LSTM	Train	MSE	59.47	70.27	65.03	50.99	59.32	61.46
		RMSE	1.40	1.53	1.47	1.30	1.40	1.43
		*p*-Value	0	0	2.32 × 10^−37^	0	0	0
		Statistic Test	−54.83	29.37	12.77	63.87	−48.26	−22.29
	Test	MSE	365.62	343.72	380.60	387.22	393.99	397.99
		RMSE	3.49	3.38	3.56	3.59	3.62	3.64
		*p*-Value	0	0	0	0	0	0
		Statistic Test	−107.44	−67.80	−43.04	20.16	−119.22	−76.89
BLSTM	Train	MSE	67.87	58.12	65.03	68.13	59.72	57.84
		RMSE	1.50	1.39	1.47	1.50	1.41	1.38
		*p*-Value	0	0	0.04	0	2.22 × 10^−5^	0
		Statistic Test	−46.73	−30.94	−2.01	38.13	−4.24	−60.83
	Test	MSE	341.95	384.18	356.68	380.71	389.80	384.17
		RMSE	3.37	3.57	3.44	3.56	3.60	3.57
		*p*-Value	0	0	0	0	0	0
		Statistic Test	−135.04	−100.03	−108.11	−34.20	−38.65	−97.62
GRU	Train	MSE	49.42	57.32	51.46	58.84	52.88	55.19
		RMSE	1.28	1.38	1.30	1.40	1.32	1.35
		*p*-Value	0	0	0	0	0	0
		Statistic Test	24.19	−16.59	−34.83	−20.20	−43.28	−108.03
	Test	MSE	365.82	427.76	414.09	360.77	401.54	380.03
		RMSE	3.49	3.77	3.71	3.46	3.65	3.55
		*p*-Value	0	0	0	0	0	0
		Statistic Test	−86.31	−67.13	−73.70	−82.58	−112.61	−161.59

**Table 3:** Robustness analysis of prediction models (LSTM, BLSTM, GRU) trained under varying levels of white Gaussian noise (SNR: 0 to 20 dB). In contrast to time series forecasting—which predicts future values based on historical data—this regression task involves estimating continuous target variables from input data at each time step without temporal extrapolation. Performance is measured using MSE, RMSE, and PCC on both training and test sets to assess each model’s sensitivity to noisy training conditions.

		Evaluation Metrics	SNR 0 dB	SNR 1 dB	SNR 2 dB	SNR 5 dB	SNR 10 dB	SNR 20 dB
LSTM	Train	MSE	5.32	8.55	9.53	6.55	22.54	12.33
		RMSE	2.30	2.92	3.08	2.55	4.74	3.51
		PCC	0.99	0.99	0.99	0.99	0.99	0.99
		*p*-Value	0.009	0.0003	0	4.55 × 10^−19^	0	0
		Statistic Test	1.65	3.59	−17.17	−8.92	−37.64	−24.79
	Test	MSE	55.60	106.38	49.31	84.61	135.79	68.58
		RMSE	7.45	10.31	7.02	9.19	11.65	8.28
		PCC	0.99	0.99	0.99	0.99	0.99	0.99
		*p*-Value	2.99 × 10^−23^	4.09 × 10^−39^	0	0	0	0
		Statistic Test	−9.93	−13.08	−16.66	−19.18	−40.00	−24.21
BLSTM	Train	MSE	16.58	20.34	5.98	13.46	8.01	13.17
		RMSE	4.07	4.51	2.44	3.66	2.83	3.63
		PCC	0.99	0.99	0.99	0.99	0.99	0.99
		*p*-Value	0.0002	0	0.02	0	1.28 × 10^−7^	1.20 × 10^−8^
		Statistic Test	3.70	28.69	−2.28	−20.26	−5.28	−5.69
	Test	MSE	84.50	57.64	46.54	68.45	60.91	63.08
		RMSE	9.19	7.59	6.82	8.27	7.80	7.94
		PCC	0.99	0.99	0.99	0.99	0.99	0.99
		*p*-Value	0	8.84 × 10^−40^	1.32 × 10^−18^	0	5.87 × 10^−26^	1.06 × 10^−21^
		Statistic Test	−14.67	13.19	−8.80	−20.50	−10.53	−9.57
GRU	Train	MSE	8.62	166.03	67.38	13.84	5.20	10.46
		RMSE	2.93	12.88	8.20	3.72	2.28	3.23
		PCC	0.99	0.99	0.99	0.99	0.99	0.99
		*p*-Value	3.45 × 10^−27^	0	0	0	0.03	0
		Statistic Test	−10.79	86.45	−18.58	16.08	2.06	−20.38
	Test	MSE	72.60	363.86	251.28	60.28	51.26	74.67
		RMSE	8.52	19.07	15.85	7.76	7.16	8.64
		PCC	0.99	0.97	0.99	0.99	0.99	0.99
		*p*-Value	0	0	0	9.34 × 10^−8^	6.75 × 10^−11^	0
		Statistic Test	−20.09	27.96	−36.78	−5.33	−6.52	−18.47

## Data Availability

The datasets generated during and/or analyzed during the current study are available from the corresponding author upon reasonable request.

## Supplementary Materials

The following supporting information can be downloaded at: https://www.mdpi.com/article/10.3390/s25113528/s1.
