# Supplementary material for: Enhancing Upper Limb Exoskeletons Using Sensor-Based Deep Learning Torque Prediction and PID Control
Source: Sensors (Basel). 2025 Jun 3;25(11):3528. doi: 10.3390/s25113528 (PMC12158260; doi:10.3390/s25113528)
Supplement: Supplementary file 1 [file sensors-25-03528-s001.zip › sensors-3635958-supplementary.pdf]

1 Supplementary

EMG signal pattern recognition is employed to identify movement patterns over time.

1.1 Root Mean Square

Reflects muscle activation level, strongly correlated with torque output.

$$RMS = \sqrt{\frac{1}{N}\sum_{i=1}^{N} x_i^2}$$

1.2 Modified Mean Absolute Value Type 1

Enhance amplitude detection by weighting signal segments differently.

$$MAV1 = \frac{1}{N}\sum_{i=1}^{N} w_i|x_i| \quad w_i = \begin{cases} 1 & 0.25N \leq i \leq 0.75N \\ 0.5 & else \end{cases}$$

1.3 Modified Mean Absolute Value Type 2

Enhance amplitude detection by weighting signal segments differently.

$$MAV1 = \frac{1}{N}\sum_{i=1}^{N} w_i|x_i| \quad w_i = \begin{cases} 1 & 0.25N \leq i \leq 0.75N \\ 4i/N & 0.25 \geq i \\ 4(i-N)/N & 0.75 \leq i \end{cases}$$

1.4 Waveform Length

Quantifies signal complexity and duration of muscle activity.

$$W = \sum_{i=2}^{N} |x(k) - x(k-1)|$$

1.5 Mean Absolute Value

Measures signal amplitude, useful for detecting muscle contraction intensity.

$$MAV = \frac{1}{N}\sum_{i=1}^{N} |x_i|$$

1.6 Zero Crossing

Indicates frequency changes, useful for detecting muscle fatigue.

$$ZC = \sum_{i=1}^{N-1} (sgn(x_i * x_{i+1}) \cap |x_i - x_{i+1}|) \geq threshold$$

1.7 Difference in Absolute Standard Deviation Value

Tracks signal variability.

$$DASDV = \sqrt{\frac{1}{N-1}\sum_{i=1}^{N-1}(x_{i+1} - x_i)^2}$$

1.8 Slope Sign Change

Tracks signal variability, related to rapid force fluctuations.

$$SSC = \sum_{i=2}^{N-1}\left[sgn[(x_i - x_{i-1}) * (x_i - x_{i+1})]\right]$$

1.9 Simple Square Integral

Represents energy content, linked to force production.

$$SSI = \sum_{i=1}^{N}|x_i|^2$$

1.10 Willison Amplitude

Counts signal spikes, indicating motor unit recruitment.

$$WAMP = \frac{1}{N}\sum_{i}^{N-1} f(|x_i - x_{i+1}|) \quad f(x) = \begin{cases} 1 & x > x_{th} \\ 0 & otherwise \end{cases}$$

1.11 V-Order

Nonlinear amplitude estimator, robust to noise.

$$v - order = \left(\frac{1}{N}\sum_{i=1}^{N} x_i^v\right)^{\frac{1}{v}}$$

1.12 Myo pulse Percentage Rate

Estimates muscle activation duration.

$$MYOP = \sum_{i=1}^{N} f(x_i) \quad f(x) = \begin{cases} 1 & if\ x > threshold \\ 0 & if\ x < threshold \end{cases}$$

1.13 Average Amplitude Change

Measures signal dynamics over time.

$$AAC = \frac{1}{N}\sum_{i=1}^{N-1}|x_{i+1} - x_i|$$

1.14 Standard Deviation

Reflects signal dispersion, related to force steadiness.

$$STD_{N-1} = \sqrt{\frac{1}{N-1}\sum_{i=1}^{N}(x_i - \bar{x})^2}$$

Notes:
N: Window Size
x_i: i the sampled EMG signal in the current window
$sgn(x) = \begin{cases} 1, & if\ x \geq threshold \\ 0, & otherwise \end{cases}$
Threshold: Noise level of the EMG signal
